# When Appearance Misleads: The Role of the Entomopathogen Surface in the Relationship with Its Host

**DOI:** 10.3390/insects11060387

**Published:** 2020-06-23

**Authors:** Maurizio Francesco Brivio, Maristella Mastore

**Affiliations:** Laboratory of Comparative Immunology and Parasitology, Department of Theoretical and Applied Sciences, University of Insubria, 21100 Varese, Italy; maristella.mastore@uninsubria.it

**Keywords:** entomopathogen surface, nematodes, *Bacillus thuringiensis*, fungi, wasps, insect immunity

## Abstract

Currently, potentially harmful insects are controlled mainly by chemical synthetic insecticides, but environmental emergencies strongly require less invasive control techniques. The use of biological insecticides in the form of entomopathogenic organisms is undoubtedly a fundamental resource for the biological control of insect pests in the future. These infectious agents and endogenous parasites generally act by profoundly altering the host’s physiology to death, but their success is closely related to the neutralization of the target insect’s immune response. In general, entomopathogen parasites, entomopathogenic bacteria, and fungi can counteract immune processes through the effects of secretion/excretion products that interfere with and damage the cells and molecules typical of innate immunity. However, these effects are observed in the later stages of infection, whereas the risk of being recognized and neutralized occurs very early after penetration and involves the pathogen surface components and molecular architecture; therefore, their role becomes crucial, particularly in the earliest pathogenesis. In this review, we analyze the evasion/interference strategies that entomopathogens such as the bacterium *Bacillus thuringiensis*, fungi, nematocomplexes, and wasps implement in the initial stages of infection, i.e., the phases during which body or cell surfaces play a key role in the interaction with the host receptors responsible for the immunological discrimination between self and non-self. In this regard, these organisms demonstrate evasive abilities ascribed to their body surface and cell wall; it appears that the key process of these mechanisms is the capability to modify the surface, converting it into an immunocompatible structure, or interaction that is more or less specific to host factors.

## 1. Introduction

In order to survive, a parasite must reach equilibrium with its host, a too efficient parasite may exterminate its host, whereas a too permissive parasite could have low fitness and lower reproduction efficiency for guaranteeing its survival [1,2]. In many cases, evolution and selection have fine-tuned host–parasite relationships to lead to long survival of the parasitized invertebrate hosts [3,4]. On the other hand, in the case of entomopathogens, the balance is shifted towards the death of the host, as the pathogen’s main objective is to exploit the host’s body, even after killing it, to complete its reproduction and life cycle.

To be successful, a foreign organism penetrating the body of an invertebrate must overcome the host immune defenses, implementing active and passive strategies effective for evading and/or depressing the host immune response.

Insects can be infected through different means: microorganisms or parasites can reach the hemocoel, or the gut, through injury, ingestion of contaminated food, damaging or infiltrating the exoskeleton, or body openings; but in all cases, the invaders are challenged by the systemic or local host immune defense. Insect immune defenses occur mainly in more body regions, such as the cuticle, the hemocoel, in which insect blood (hemolymph) flows, and the gut, which is colonized by endogenous bacteria (microbiota).

The present review outlines how the cellular and body surfaces of organisms commonly used as biological control agents, such as the bacterium *Bacillus thuringiensis* (Bt), entomopathogenic fungi (EPF), entomopathogenic nematodes (EPN), and wasps, are central to their success in killing the hosts. We describe in detail the role played by these pathogens’ surface molecules and molecular complexes as key elements in preventing immune recognition, and therefore the activation of molecular switches that control the triggering of effector processes. We also outline the contribution of secretions and secondary metabolites released during the infection and that are involved in the immunological interference.

### 1.1. An Overview of Insect Immunity

Although insects lack the anticipatory immune responses and immunoglobulin molecules [5,6,7,8] typical of vertebrate immunity, they are capable of mounting highly efficient cellular and humoral defenses [9,10,11,12,13]. Insect innate immunity is based on the interaction of typical non-self molecules termed PAMPs (pathogen-associated molecular patterns), with their receptors, termed PRRs (pathogen recognition receptors) circulating in body fluids or exposed on immunocompetent cell membranes. An important feature of PAMPs is their strongly conserved structure, which is invariant between the organisms of a given class [14,15].

Typical PAMPs are peptidoglycans (PGN), lipopolysaccharides (LPS), sugars such as beta-glucan (beta-Glu), various proteins, glycoproteins, lipids, and distinct nucleic acid motifs that are unique and essential for a microorganism’s survival.

These compounds are recognized by specific receptors, such as PGN receptor proteins (PGRP), LPS-binding proteins (LBP); sugar-binding proteins (SBP), and the cellular PRRs, i.e., the Toll and Imd receptors [15,16,17,18,19,20].

**Figure 1:**
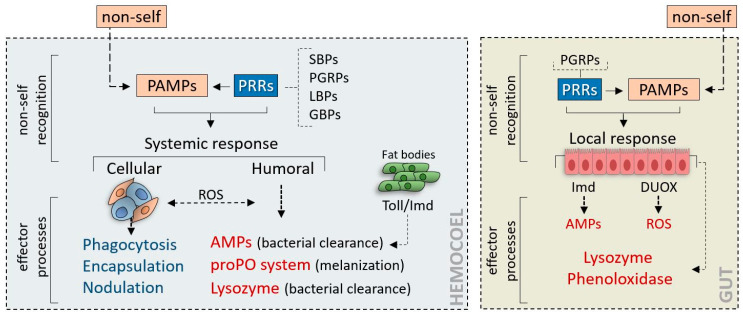
The insect immune system. Insect innate immunity is based on the recognition of non-self by cellular and soluble host receptors; the recognition of foreign molecules (PAMPs) by host receptors (PRRs) initiates a synergistic network of cellular and humoral effector responses aimed at neutralizing the non-self. As in vertebrates, the innate immune response in insects can be triggered systemically (hemocoel, left) and locally, as in the gut (right). Unlike systemic innate immunity, where the Toll and Imd pathways are essential, the immune response in the gut mucous membrane epithelium depends on the Imd pathway and on the DUOX pathway. SBPs: sugar-binding proteins; PGRPs: peptidoglycan receptor proteins; LBPs: lipopolysaccharide-binding proteins; GBPs: glucan-binding proteins; proPO: prophenoloxidase-phenoloxidase; AMPs: antimicrobial peptides; ROS: reactive oxygen species; DUOX: dual oxidase.

The effector defense processes are typically triggered when the interaction between PAMPs and PRRs is performed correctly; when the host receptors cannot interact by specifically recognizing their pathogen counterparts, the non-self is not identified, and the subsequent immune processes are not activated. In this case, the pathogen may be able to overcome the host immune defenses and thus carry out its lethal action (Figure 1).

The molecules and molecular complexes involved in the humoral defense reactions include the prophenoloxidase–phenoloxidase (proPO) system, lysozyme, and antimicrobial peptides (AMPs) [21,22,23]. proPO system–mediated melanin deposition around foreign bodies is usually observed during cellular and humoral encapsulation of foreign bodies. The proPO system, which is activated by invading microorganisms or parasites, is a complex enzyme cascade in which the last active enzyme (phenoloxidase) oxidizes phenols into quinones, and these compounds will then autocatalyze into melanin [24]. This system is also a key element in foreign body recognition and opsonic factors production [25]; it is now considered to represent an integral component of insect immunity; moreover, the proPO activation cascade shows similarity with the human complement system [23]. The lysozyme, present in many body regions, also contributes to the neutralization of bacteria; lysozyme catalyzes the hydrolysis of 1,4-beta-linkages between *N*-acetylmuramic acid and *N*-acetyl-D-glucosamine residues in PGN, which is the major component of the Gram-positive bacterial cell wall [26]. AMPs are members of inducible humoral components, as insects produce them after exposure to bacteria or fungi; their role is microorganism clearance: these molecules are coded by fat body cell genes that are activated via the intracellular transduction pathways triggered by the Toll and Imd receptors [10,27,28]. In general, in all immune processes, the humoral components cooperate synergistically with immunocompetent cells. Cellular immune responses are carried out by several types of circulating immunocompetent cells (hemocytes); the cellular processes include phagocytosis, encapsulation, and nodule formation [29,30,31,32]; the type and intensity of the reactions depend on the size, number, and characteristics of the foreign body.

Locally, the midgut protects its microbiota from pathogens ingested during feeding by means of humoral responses [33]. The midgut immune system can specifically neutralize and eliminate pathogens with minimal interference to commensal and mutualistic bacteria.

During gut infection, the gut epithelial cells trigger various intracellular pathways, some of which (e.g., DUOX (dual oxidase)) promote a cascade of reactive oxygen species (ROS); moreover, the Imd pathways can be activated for local AMP synthesis, playing additional roles in the control of ROS-resistant pathogens [33,34].

### 1.2. Strategies of Parasites and Microorganisms

**Figure 2:**
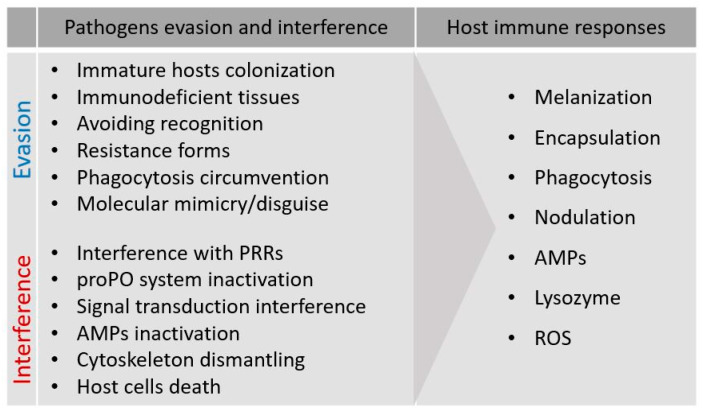
Summary of pathogens immune evasion/interference strategies and host immune responses.

Parasites or microorganisms may successfully colonize hosts by evading immune recognition and thus preventing the effector mechanisms; circumvention of the host immune system can be achieved by molecular mimicry/disguise strategies, or by colonizing young hosts and host tissues with low immunocompetence (passive strategies) [1,35] (Figure 2). Alternatively, or concurrently, many parasites can depress either cell-mediated or humoral effector mechanisms by active processes that affect host immunity (interference) [36,37].

Molecular mimicry is a passive strategy by which parasites become antigenically related to the host and thus avoid evoking host immune responses; true molecular mimicry can be defined as the endogenous production of mimicking molecules that are usually exposed on the invader’s body surface or cell surface. Despite the appeal of molecular mimicry as a mechanism of immune avoidance and the identification and characterization of many antigens shared between invertebrates and vertebrates, as major histocompatibility complex (MHC) antigens, oligosaccharides, blood glycolipids, tropomyosin, and alpha-macroglobulin [38,39,40], few studies on invertebrate pathogens have investigated the protective effect of shared antigens [41,42].

Molecular disguise, another form of mimicry, is described as the acquisition (sequestering) of molecular components from the host [1,43,44] to form a body coating antigenically related to the host [45,46,47,48,49]. A Strepsiptera (*Stichotrema dallatorreanum*) implements an alternative disguise mechanism, using the host epidermal tissues to wrap itself in a sort of bag that camouflages the endoparasite, making it immune to the recognition of the insect [50]; this strategy allows the parasite to carry out the necessary trophic exchanges within the host body that are essential for its development and reproduction.

The stage of host development is also essential for determining the outcome of parasitization [51]; in general, early-instar insect larvae show reduced immune activity, often due to lower hemocyte number or a different array of cell populations [52,53]; consequently, parasites that penetrate young hosts can find a more favorable environment to overcome host defenses. Moreover, as a further passive strategy, some parasites can colonize low-reactivity host tissues [54]. An example is represented by some wasps that lay their eggs, with surgical precision, in the host nerve ganglia into which hemocytes do not normally circulate, so the wasp embryo can develop unmolested within the host [55].

As mentioned above, active strategies can be referred to as interference; in this case, parasites show aggressive suppression or alteration of the host immune system defenses. Interference can be directed against host humoral factors that are neutralized by the parasite, or against immunocompetent cells that could be targeted [1,56,57]. A common type of humoral depression involves the proPO system, one of the main targets for many nematodes, wasps, or microorganisms; this is probably due to the need to neutralize its drastic effects, which are triggered quickly in the presence of an infection. The interaction between PAMPs and the Toll/Imd cell receptors is also a target of pathogen strategies, as hampering intracellular pathway activation prevents the synthesis and release of AMPs [58,59].

Many active strategies consist of drastic actions directed against immunocompetent cells, particularly hemocytes, fat body cells, and in some cases, the gut epithelial cells. Several pathogen cytotoxic molecules, either released or on the surface, can interfere with immunocompetent cell functions, inhibiting adhesion, altering cytoskeletal protein assembly, damaging the plasmalemma, and inducing cell death [37].

We must consider that the earliest interaction of the host immune system will occur with the invading organism’s body (or cell) surface; therefore, the phase of immunological recognition and thus the possible activation or failure of the immune defenses, is strongly dependent on the involvement of the invader’s surface molecules and molecular architecture.

## 2. Entomopathogens

### 2.1. Bacillus thuringiensis

A spore-forming Gram-positive bacteria, the Bt is considered a useful biopesticide; this soil-borne entomopathogenic bacteria is the most studied and known of the microbial insect control agents currently available; its host spectrum comprises a broad range of arthropods [60]. Bt is part of the *B. cereus* group that includes species capable of forming highly resistant dormant endospores in response to harsh environmental conditions [61,62,63]. Once Bt spores enter the host organism, their huge arsenal of virulence factors allows them to migrate from the gut to the hemolymph, where they turn to the vegetative phase, propagate, and disseminate within the host organism. After the host dies of resulting septicemia, the bacteria dwelling in the corpse propagate until they exhaust all consumable organics, and then transit to the sporulation phase [64].

To cause infection in insect hosts, Bt must be ingested and must overcome various physical barriers, such as the gut peritrophic membrane, epithelium, commensal microbiota, and peristalsis, as well as the chemical defenses present in the digestive system, such as pH and proteases other than local AMPs. Commensal microbiota provides a competitive environment for Bt settlement and also produces antimicrobial compounds that could hamper the action of possible pathogens [65]. After overcoming these defenses, Bt must address both the hemolymph cellular and humoral defenses to achieve its goal.

**Figure 3:**
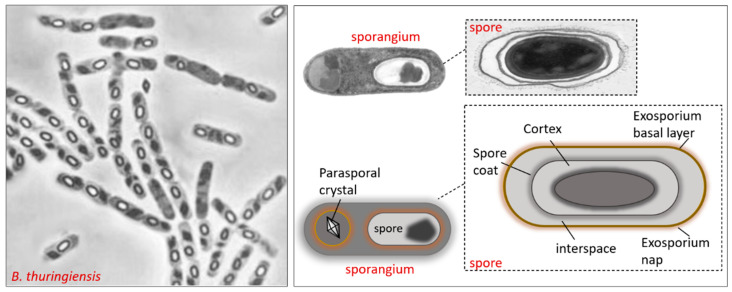
*B. thuringiensis* sporangium containing the spore and parasporal inclusion with toxins crystal. The spore and paraspore are protected by the presence of the exosporium during interaction with the host midgut environment.

The mechanism of Bt pathogenesis against insects is a complex process that involves various components; to counteract the host immune system, the bacteria utilizes a wide variety of virulence factors, such as molecular structures and biochemical systems, which help the bacteria cause physiological disorders [60,66,67]. Bt toxins are generally produced during infection, and most of them interact with the immune system, either directly, as immune cells are their primary target, or indirectly, affecting the gut local responses [68]. As mentioned above, the Bt life cycle is characterized by two phases: vegetative cell division and spore development; the vegetative phase is characterized by rod-shaped cells and generates two similar daughter cells, while sporulation comprises asymmetric cell division, in which various stages of the cells, called sporangium, are observable. Between stage III and VI, forespore and parasporal crystals are formed and surrounded by the exosporium, cortex, and spore coat [69,70]; the mature spore and parasporal crystals are liberated by lysis of the sporangium (Figure 3).

When spores and paraspores are in the host larvae gut, the outer shell of the spore remains intact while the paraspore matrix dissolves. Parasporal crystals, which are toxic to the larvae, consist of one or more proteins (Cry (crystal) and Cyt (cytolytic) toxins, also termed delta-endotoxins); Cry and Cyt are parasporal protein inclusions: Cry proteins exhibit toxic effects on the target organism and Cyt proteins exhibit cytolytic activity. These toxins are highly harmful to many insects, but are safe for humans, vertebrates, and plants [71,72].

Mature spores represent a cryptobiotic state of the bacteria, and are resistant to environmental stress and indefinitely viable, so that, under favorable environmental conditions, such as the hemocoel of insects, they germinate in the vegetative cells. Endospore formation is a survival mechanism rather than a reproduction mechanism. During sporulation, an external protein layer (spore coat) forms around the spore and paraspore; after entry and before the lethal action of Bt toxins, spores and paraspores meet the intestinal milieu and must counteract the local immune defenses such as ROS and AMPs; in this phase, the surface of both the spore (exosporium) and paraspore play a key role in their protection [73,74,75,76]. The exosporium defines the limit between the spore (or paraspore) and the host midgut environment with which it interacts; this interaction represents the first point of contact of the spore with the host gut cells [77]. The exosporium surface helps Bt to adhere to host tissues; it contains enzymes that control spore germination and protect the spores from elimination by hemocytes [77,78]. This structure is composed of proteins, lipids, and carbohydrates, and contains at least one paracrystalline layer, termed the basal layer, which has arrays of crown-like structures that may also act as a substrate for the binding or adsorption of other proteins, as well as a scaffold to which the hairy nap attaches [78,79,80,81]. The exosporium is separated from the spore coat by a region known as the interspace and is the final layer of the spore to be assembled [75,82,83]. The exosporium consists of a hair-like outer nap and a paracrystalline basal layer, and contains several proteins that are deposited around the spore [84,85,86,87,88,89]; nap filaments are formed by oligomers of the glycoprotein BclA, a collagen-like protein involved in the first interactions with the host surface [90,91].

The enzymes associated with the exosporium include alanine racemase, inosine hydrolase, and superoxide dismutase; these enzymes may be involved in preventing premature germination and in protection against cellular immunity through the detoxification of superoxide free radicals [78,92,93].

The role of exosporium proteins was investigated by means of protein deletion and immunolabeling within the structure; in some *Bacillus* species, the presence of a protein named YwdL appears to increase the hydrophobic properties of the exosporium compared to spores that lack the protein [85,94].

Only a few reports attempted to explain the mechanisms involved in the interference between Bt and the host insect immune defenses [95,96]. However, Bt overcomes the immune defenses by suppressing the humoral immune system; the analysis of *Plutella xylostella* immune gene expression, after exposure to Bt at different times [97] showed a marked reduction in the lepidopteran’s immune response. Li and coworkers [97] showed that, in the presence of the bacteria, many more immune genes were downregulated than upregulated. In the presence of Bt, PRRs such as PGRPs, beta-glucan–binding proteins (GBPs), and scavenger receptors were downregulated, except for some members of the lectin family, which were upregulated. The control of expression occurs either during the recognition phase or during the activation of signal transduction pathways that play an essential role in triggering the immune and physiological processes [97,98]. Up- or downregulation of serine protease expression, as well as of the components of the Toll pathway (Spätzle, MyD88, cactus), was observed in response to the bacterium [99] (Ross et al., 2003). Crava et al. [100] analyzed the regulation of AMP and lysozyme gene transcription in the midgut of *Spodoptera exigua* larvae after sublethal oral intoxication with Bt virulence factors; the results showed copious transcriptional midgut response of AMPs when Bt spores were ingested, suggesting a protective role for the exosporium during the pathogenic phase of Bt.

### 2.2. Entomopathogenic Fungi

EPF are another class of bioinsecticides, and even though more than 700 species are known, about 80% of commercial formulations are based on the genera *Metarhizium* and *Beauveria* [101]. In nature, EPF are natural regulators of insect populations, and the features of their life cycle make them useful mycoinsecticide agents against insect pests and human disease vectors [102,103]. EPF colonization of insect hosts comprises different steps: adhesion to the insect exoskeleton (conidia and blastopores), germination, development of infectious structures (appressoria and pegs), access to the host hemocoel and proliferation, and finally, sporulation after release from the insect corpse [104,105].

**Figure 4:**
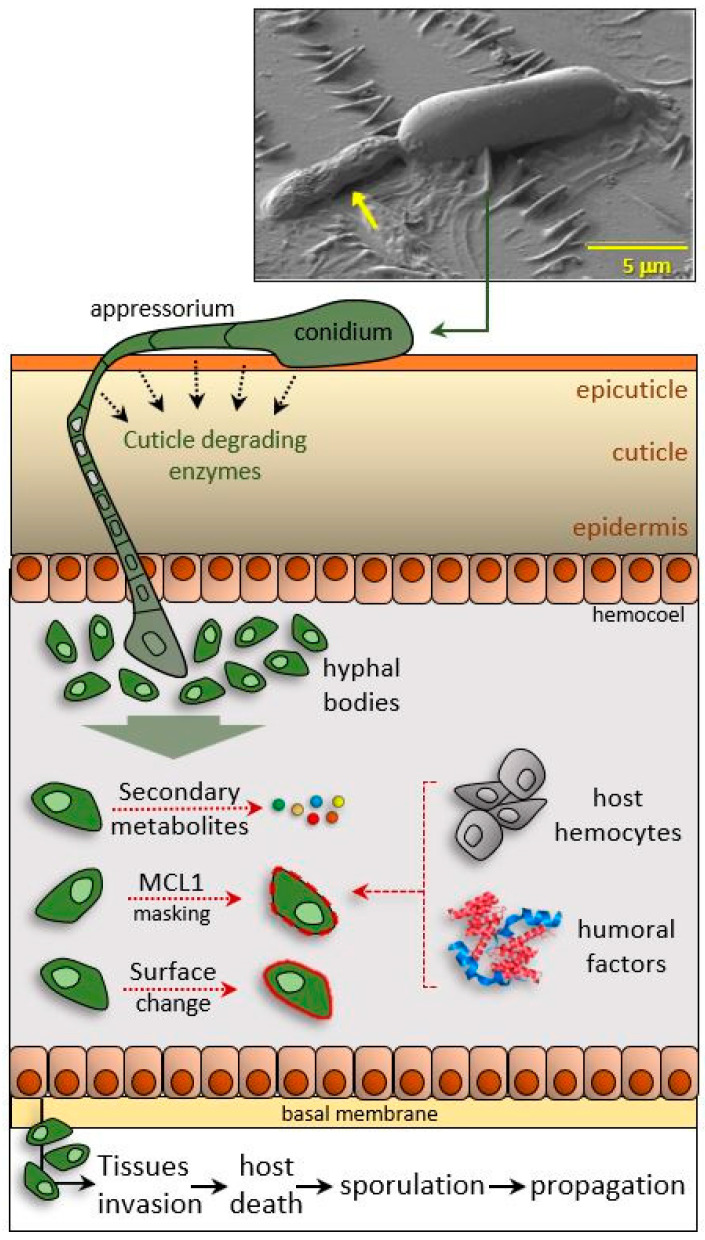
Infection strategies of entomopathogenic fungi. EPF penetrate the insect exoskeleton by means of structures as the appressorium, then hyphal bodies release secondary metabolites and, after surface changes, avoid host immune recognition. SEM micrograph was kindly provided by N.A. Ratcliffe.

EPF infect their hosts by penetrating the cuticle and gaining access to the hemolymph; in the hemocoel, under favorable conditions, they grow by utilizing the nutrients present in the hemolymph. Once inside the insect body, EPF face a series of potent immune responses; EPF have evolved multiple strategies for circumventing and neutralizing insect immune defenses; these include both escaping mechanisms that involve their wall, and interference mechanisms with the host immune response, carried out by released secondary metabolites and secretions (Figure 4).

EPF have evolved evasion strategies that involve changes in cell wall composition; these adaptations modify surface components that are typically recognized by the host immune system (cell wall remodeling), thus allowing hyphal bodies to circulate undisturbed in the hemolymph. The carbohydrate moiety of the EPF surface depends on the genus, and carbohydrates are the main compounds commonly recognized by host PRRs for triggering immune signaling cascades [106], so their presence and structure are responsible for their discrimination as non-self.

Unlike fungal conidia or hyphae, hyphal bodies seem to have fewer sugar epitopes, which allow them to avoid recognition in the hemolymph [106,107,108].

In the host hemolymph, *Beauveria bassiana* grows as single yeast-like cells with very thin cell walls; blastospores isolated from the hemolymph show carbohydrate epitope shielding that protects the cells from immune recognition [106,109,110,111]; moreover, the shift to blastospores reduces the number of PAMPs on the cell surface, reducing the effectiveness of recognition by the host PRRs [112].

Cell wall proteins, such as CWP10 or MAD1, are also involved in the mechanisms of escape, playing a critical role in the mechanisms of adhesion to the insect exoskeleton [113,114,115]; besides, in *Metarhizium anisopliae* MCL1, a collagen-like protein coded by the *Mcl1* gene and present on the surface acts as an antiadhesive protective coating to mask antigenic cell wall beta-glucans, preventing the recognition of hyphal bodies by the host hemocytes, avoiding encapsulation and nodulation [116,117]. However, the molecular bases of these modifications have not been fully clarified and the extent to which they reflect de novo protein synthesis, or the morphological and topological rearrangement of cell surface components, is unclear [117,118].

In addition to the surface evasion mechanisms, EPF have also evolved immunodepressive strategies in which secondary metabolites and secretions lead to immunological alterations and a severe physiological disorder that induces a lethal pathological condition in the host. Throughout infection, EPF synthesize and secrete a wide range of bioactive compounds, including bassianin, bassiacridin, oosporeins, cyclosporine, and destruxins [119,120,121]. Some of these metabolites are responsible for EPF virulence and host specificity and can suppress the host immune response [122,123]. For example, *Metarhizium* destruxins can inhibit the expression of genes encoding AMPs and can block phagocytosis by inhibiting V-ATPase (V-type ATPase) [124]. Oosporein produced by *B. bassiana* inhibits proPO activity and downregulates expression of the antimicrobial gallerimycin in *Galleria mellonella* larvae [121,125]. Moreover, some *Metarhizium* and *Beauveria* strains are resistant to the antifungal peptide drosomycin, whose synthesis is regulated by activation of the Toll pathway [126,127]. During the penetration phase, most EPF produce proteases (e.g., Pr1) to degrade the cuticle and activate the proPO pathway, although some EPF can reduce insect PO activity by suppressing protease activity [128]. Recently, it was reported that *Beauveria bassiana* can interact with gut microbiota to accelerate mosquito death via downregulation of local AMPs [103].

### 2.3. Entompathogen Wasps

Entomopathogen wasps are a large group in the Hymenopteran superfamily and are used as effective bioinsecticides against different insect orders, such as Lepidoptera, Coleoptera, Diptera, and Hemiptera. Wasps lay their eggs in the body cavity of their host, where their progeny feed, and permissive hosts die because of wasp development. Many wasp species successfully parasitize one or a limited permissive host species [129,130,131,132,133].

**Figure 5:**
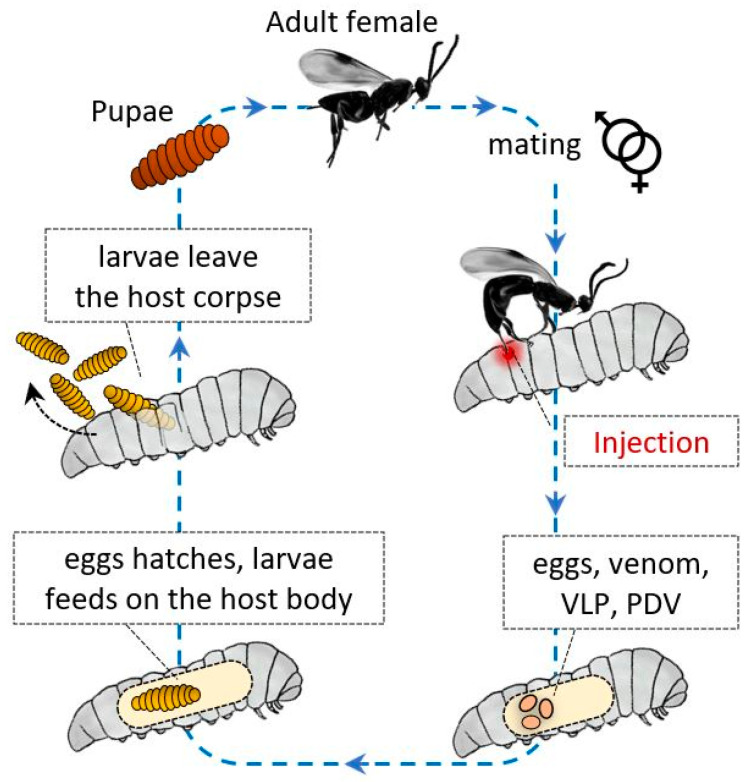
Some entomopathogen wasps reproduce sexually, then when they find a host, the females inject eggs into it; together with the gametes, they inject polydnavirus (PDV), virus-like particles (VLPs), and venom compounds. The compounds and viruses help the egg to not be recognized by the host immune system. In this manner, the eggs can hatch, and the larvae can develop undisturbed by feeding on the corpse of the parasitized insect.

These Hymenoptera have evolved a variety of strategies to avoid host immune responses, and as described for other parasites and microorganisms, these strategies can be divided into passive and active mechanisms. In general, factors of maternal and embryonic origin protect their progeny from the host humoral and cellular immune system responses (Figure 5).

After injection, the wasp eggs and developing larvae are supported by various factors, and protective active strategies are carried out by various components, such as polydnaviruses (PDVs) and glycoproteins similar to PDVs, termed virus-like particles (VLPs) [134,135,136,137]. The eggs are injected together with calix fluid, which contains serine protease inhibitors that can inactivate the cellular response of hemocytes [138] and the proPO system [139]; besides, venom components can be coinjected, activating intracellular caspases and inducing apoptosis in the plasmatocytes and lamellocytes of *Drosophila melanogaster* [140]. All these components can avoid and deactivate the recognition processes, neutralizing the host immune response.

Concerning passive evasion strategies, ichneumonid wasps can implement egg and larval surface protection by means of covering factors [141,142]; these factors do not appear to affect cellular responses, as the host is able to react to wounds with encapsulation processes; in the absence of these protective compounds, wasp eggs are encapsulated, probably due to reactivity against some chorion proteins. The authors demonstrated that the egg surface layers of *Cardiochiles nigriceps* protect the egg, avoiding recognition by hemocytes; the data suggested that the glycoprotein fibrous layer present on mature eggs of the wasp helps to evade encapsulation by *Heliotis virescens* hemocytes.

VLPs produced in the calyx of *Venturia canescens* play a main role in immunoevasion by the wasp, as they cover the egg with a coat immune compliant with the host [135]. VLPs can be considered factors involved in passive evasion [143], even though further studies have identified several VLPs that appear to modulate the host immune physiology [144,145,146].

A glycoprotein called hemomucin plays a central role in passive evasion [147,148]. Hemomucin is an *O*-glycosylated protein identified on the surface of eggs [148]; it shows specific affinity for hemolymph lipophorin and can form complexes with the lipoprotein that covers the egg; the complexes formed between surface mucin and lipophorin seem to avoid hemocyte recognition, as host cells are unable to adhere to surfaces coated with these molecular complexes [149,150]. Hu et al. [148] demonstrated that *O*-glycosidase treatment or antibody binding of hemomucin resulted in the loss of protection and encapsulation of wasp eggs.

**Figure 6:**
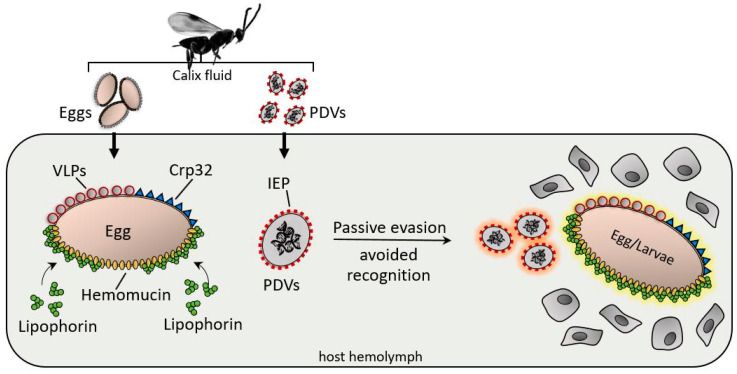
After injection, various surface factors protect the wasp eggs and PDV from the host immune system. Virus IEP (immunoevasive proteins), a thin-layer component covering PDVs; hemomucin, an *O*-glycosylated transmembrane protein with affinity for host hemolymph proteins (e.g., lipophorins); Crp32, an ovarian component; and VLPs are responsible for immunoevasion strategies, protecting the PDV eggs, and larvae from hemocyte-mediated encapsulation processes.

As previously suggested, wasp eggs and larvae are often covered by surface factors that are not recognized as foreign; consequently, hemocytes do not adhere [151,152] (Figure 6).

*Cotesia rubecula* ovarian proteins have been identified as being clearly involved in passive evasion mechanisms against *Pieris rapae* immune recognition; in particular, a 32-kDa protein from the ovarian calix shows protective properties for eggs and PDVs [152,153]. Assays have demonstrated that Sephadex beads coated with a recombinant egg surface protein (Crp32) were not encapsulated when injected into hosts, whereas uncoated beads were rapidly encapsulated [154].

Passive evasion mechanisms also involve PDV surfaces; indeed Tanaka et al. [155] identified a protein (IEP) with immunoevasive properties. IEP coats the PDV particles of *C. kariyai* (CkPDV), protecting the virus from encapsulation by the hemocytes of *Mythimna separata*; the immunoevasive protein is expressed in both the venom and oviducts, suggesting a relationship in the evasion strategy between wasp tissues [156].

Host immunodepression is mainly carried out by compounds present in the venom, a complex mixture of protein and nonprotein molecules injected into the host during oviposition by the female wasp. Studies on venom composition have led to the identification and functional characterization of several compounds involved in the immune modulation of the host. Even if most of them have not been functionally characterized, it is supposed that they could be involved in venom homeostasis, transient paralysis, cytotoxicity, and hemocyte inactivation. A well-studied example is *Pimpla hypochondriaca* venom, which consists of several enzymes, protease inhibitors, neurotoxin-like, and inhibitors of hemocyte aggregation. Venom compounds from *Leptopilina boulardi*, such as the proteins RhoGAP and LbSPNy, lead to suppression of *Drosophila* host cellular encapsulation and melanization, respectively [157]; moreover, calreticulin from *C. rubecula* venom is responsible for hemocyte inactivation [158]. A further depressive mechanism was described in the eggs of several entomopathogen wasps, which upon hatching, produce and release cells called teratocytes that are responsible for immunodepression. For example, along with other maternal factors, *Cotesia plutellae* teratocytes play a role in depressing host immunity; these cells synthesize and secrete immunodepressive factors inhibiting the nodulation processes in the lepidopteran *P. xylostella* [159]. Besides, these cells are also known for their trophic functions and for altering both host development and metamorphosis [160,161].

### 2.4. Entomopathogenic Nematocomplexes

**Figure 7:**
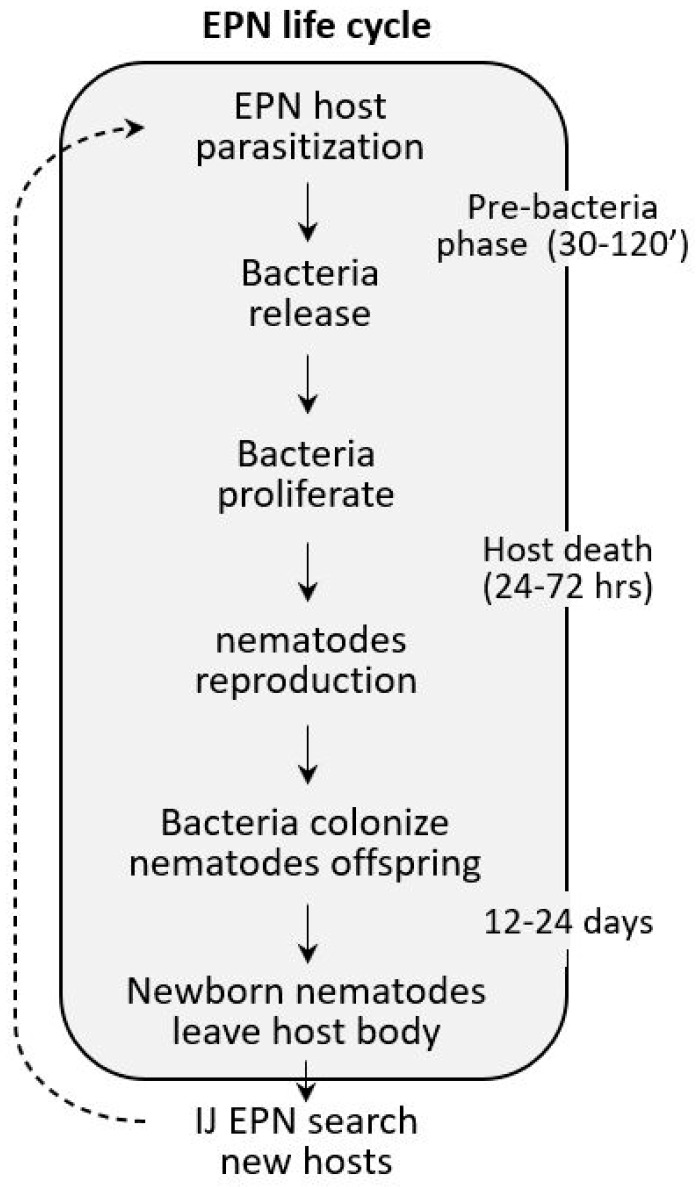
Entomopathogenic nematode (EPN) life cycle. During the IJ3 stage, the nematode penetrates the host; after 30 min to 2 h, symbiont bacteria are released in the hemolymph, bacteria proliferate, and the insect dies within 24–72 h. After reproduction, nematode offspring feed on the corpse and finally leave the host and search for a new target.

EPN used as bioinsecticides belong to the families Steinernematidae and Heterorhabditidae (Nematoda, Rhabditidae) [162,163]. Most of these nematodes, among which are *Steinernema feltiae*, *S. carpocapsae*, and *Heterorhabditis bacteriophora*, have a species-specific mutualistic relationship with bacteria; particularly, *Xenorhabdus* spp. are associated with Steinernematidae and *Photorhabdus* spp. are associated with Heterorhabditidae [164]. At the infective third stage, EPN penetrate the target insect, and after a period varying from 30 min to 2 h, release the symbiont; the bacteria proliferate and kill the host within 24–72 h. The nematodes reproduce, and the offspring grow on the corpse of the host. At 12–24 days after infection, infective third stage EPN, after swallowing the symbionts, leave the insect body and begin the search for new hosts to colonize (Figure 7).

After overcoming external barriers such as the exoskeleton, and reaching the hemocoel, EPN in the IJ3 must elude the host recognition system and/or depress the immune effector processes [165,166]. However, it is important to underline that the immunoevasive or immunodepressive processes depend on the relationship between the parasite and a specific host insect. EPN can be considered entomopathogen whose behavior is characterized by the lethal nature of their interaction with their host. It is important to remember that the success of EPN results from the cooperation between the nematode itself in the early stages of infection and the lethal action of the symbiont bacteria released at a later stage.

The symbionts are considered the main executors of the death of the host; they live in the nematode gut, and after parasitization are regurgitated in the host hemolymph. The bacteria also promote permissive conditions for EPN reproduction, supplying nutrients and affecting the proliferation of other microorganisms in the host hemocoel by producing AMPs [67,167].

We may consider EPN ticking poison bombs, which detonate, releasing the killer bacteria 60–120 min after accessing the hemocoel of the target insect. The two main strategies by which EPN avoid and counteract host immunity are molecular mimicry/disguise and interference processes. The latter strategy is achieved by means of excretion/secretion factors synthesized and released by the nematodes and symbionts; these compounds interfere with and neutralize the humoral and cellular effector processes elicited by the host in response to the infection [49,168,169]. To elude recognition, the envelope of the bomb, i.e., the nematode surface, must be considered as self by the host immune system; if these conditions occur, in the next phase, the compounds produced by the nematode and released into the hemolymph depress the immune response by interfering with and inhibiting both the humoral and cellular effector processes triggered by the host.

However, shortly after penetrating the host hemocoel, the nematode interacts with the systemic immune processes through direct interaction via its body surface [170,171], i.e., of the cuticle and the compounds present outside the cuticle (i.e., the epicuticle). These acellular structures have a peculiar molecular architecture: in the nematode phylum, the cuticle has a shared structure with variations in composition depending on the species, stage of development, and environment in which the nematode is present [172]. In general, the nematode cuticle is a protein outer covering with a small amount of lipids and carbohydrates; the internal layers consist of collagen-like proteins, while noncollagen insoluble proteins (termed cuticlins) and nonstructural proteins are present in the epicuticular cortex and exposed to the outer environment, and to the hemolymph fluid [173].

**Figure 8:**
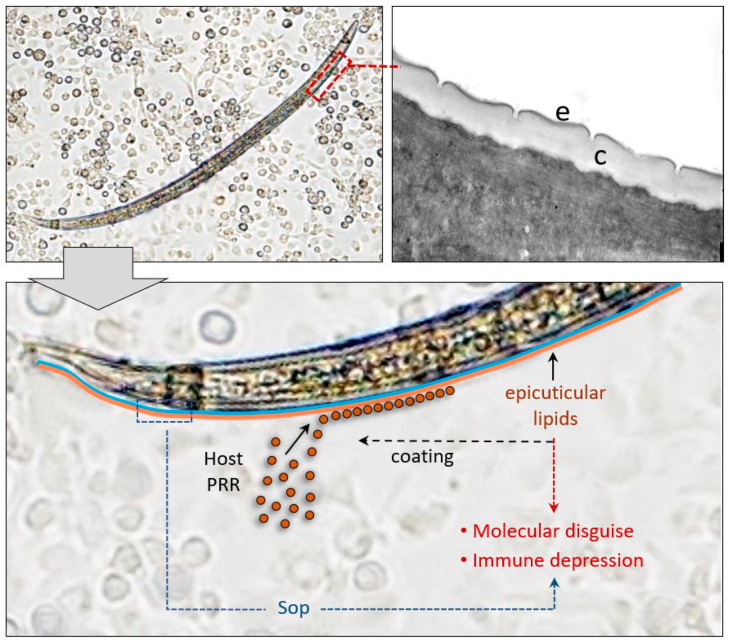
The body surface of *Steinernema* spp. interacts with hemolymph components through specific adhesion; this interaction leads to the removal of key factors fundamental for triggering the insect immune processes (as observed in *S. feltiae*); moreover, the hemolymphatic proteins attached to the epicuticle covering the nematode are not recognized by the host hemocytes (as observed in *S. feltiae* and *S. carpocapsae*).

The primary role of *S. feltiae* and *S. carpocapsae* surfaces [174] is being responsible for the immunoevasion processes; both EPN are not recognized by hemocytes, so they avoid cellular encapsulation. Moreover, the *S. feltiae* cuticle shows strong affinity for hemolymph factors, and the specific interaction of epicuticular lipids results in selective subtraction of host PRRs (HiPs), leading to general immune suppression, and the coating of the parasite with host compounds is responsible for its mimicry properties [48,175]. The mimicry properties of *S. feltiae* cuticular lipids (PCls) were also demonstrated by in vitro assays; after purification, lipids were linked to agarose microbeads; the coating rendered the microspheres, which are normally encapsulated, nonantigenic for the host hemocytes [48] (Figure 8).

Even though they are probably involved in evasion of cellular encapsulation, *S. carpocapsae* surface proteins (Sop) isolated by high salts extraction did not show significant effects on hemocyte viability, phagocytosis ability, and proPO system activity [176].

In addition to the cuticular lipids of *S. feltiae* and the coat protein SCP3a of *S. glaseri* [177] showing immunodepressive effects, active interference with the host immune processes are generally caused by molecules that are secreted or excreted (ESPs) by EPN. Many molecules have been identified as being secreted in various EPN, and all of them affect host immunity in different ways; among them, serine proteases and protease inhibitors are more represented; moreover, other proteases such as metallo-, aspartic, and cysteine proteases have been identified [126,178].

Balasubramanian et al. [179] showed that the infective *S. carpocapsae* secretes within a pool of ESPs a 29-kDa trypsin-like serine protease; in vitro tests showed that the protease significantly reduces the activity of *G. mellonella* phenoloxidase; moreover, the molecule affects the spreading ability of hemocytes, dismantling the cytoskeleton architecture of the cells. The authors demonstrated that *G. mellonella* hemocytes were unable to encapsulate *S. carpocapsae* and *S. glaseri*, unlike *H. bacteriophora*, which was recognized as foreign and encapsulated by the hemocytes.

Recently, Chang et al. [180] used mass spectrometry to identify and characterize 266 ESPs from *S. glaseri* infective juveniles; comparing these ESPs with those previously identified in *S. carpocapsae*, they identified a set of 52 proteins in the ESPs of both species. These ESP pools included tissue-damaging and immunomodulating factors, suggesting that they comprise both a pool of effectors and a specialized group of molecules directed to different target hosts.

As described for *Steinernema* spp., *H. bacteriophora* and *H. marelatus* also seem to be able to elude hemocyte recognition and encapsulation in *Tipula oleracea*, *Popillia japonica*, *Cyclocephala borealis*, and *Leptinotarsa decemlineata* [56,181,182,183], even if *L. decemlineata* hemocytes are able to encapsulate *H. bacteriophora* 15 min after its entry [184,185].

**Figure 9:**
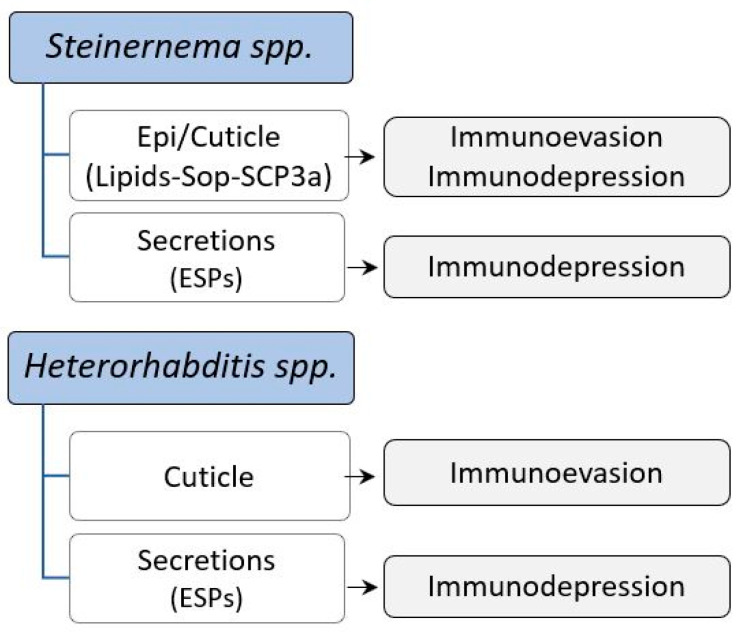
A schematic of the effects of EPN body surfaces and secretions on the immune responses of target insects.

Studies on the specific factors secreted by *H. bacteriophora* are essentially based on genomics and transcriptomics, and highlighted the modulation of genes encoding metalloprotease, chitinase, enolase, C-type lectin, and catalase, which could play a role in host immunomodulation; perhaps the products of these genes contribute synergistically to the success of the nematode, but their molecular identification would contribute to better understanding of their function (Figure 9).

Better characterization of the abovementioned gene products was achieved in *H. bacteriophora*; the EPN releases a protease affecting the cecropin B activity of *G. mellonella* [180]. Besides, as demonstrated recently, a secreted protein pool inhibits the expression of the AMP-encoding gene diptericin, interfering with the host Imd pathway [186].

**Figure 10:**
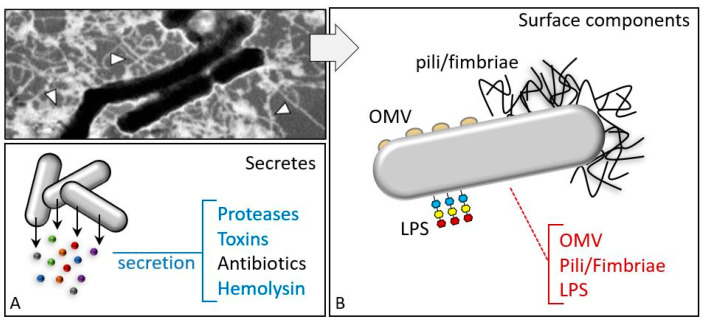
EPN bacterial symbionts have surface components and secretions responsible for evasion/depression strategies. (**A**) Secreted factors, such as proteases, toxins, and hemolysins, interfere with the host immune responses, while antibiotic compounds are synthesized and released to prevent the growth of possible competing bacteria. (**B**) Surface components and secretions from entomopathogenic bacteria, lipopolysaccharides (LPS), pili/fimbriae components, or outer membrane vesicle (OMV) content and membrane components are responsible for various immunodepression and immunoevasion processes. Transmission electron micrograph shows *X. nematophila* surface structures after negative staining.

As noted, EPN have a population of symbiotic bacteria in the intestine; particularly, *Xenorhabdus* spp. colonize a modified ventricular part of the intestine, instead *Photorhabdus* spp. are found throughout intestinal lumen. Upon penetration of the host hemocoel, the nematode ingests hemolymph, and this triggers the release of the symbionts. The bacteria, like the nematode in the early phase of parasitization, must counteract the host immune defenses, so they have developed some strategies for evading and neutralizing them. *Xenorhabdus* spp. and *Photorhabdus* spp. exist in two pleomorphic forms, usually referred to as phase I and II; phase I variants have different physiology and morphology with respect to phase II bacteria [187]. Phase I bacteria, i.e., the virulent phase, show high motility and surface molecular structures such as LPS, pili/fimbriae, flagella, and outer membrane vesicles (OMV) containing virulence factors [188,189,190,191] (Figure 10).

Various components of these structures, particularly of the pili/fimbriae, interact with the host defenses to avoid hemocyte recognition, and affect the phagocytosis and nodulation processes. Flagella promote adhesion and motility, helping host tissue colonization, while LPS affect various defense processes such as the AMPs pathways, and hemocyte recognition and viability [57,192,193].

Both *Xenorhabdus* spp. and *Photorhabdus* spp. can produce and secrete molecules, such as hemolysin, lipase, and metalloprotease. These factors play a role in cell lysis, bacterial motility, immunoprotein degradation, host cell viability, eicosanoid pathways and thus encapsulation, phagocytosis, and nodulation; other than that, they interfere with melanization processes and antimicrobial pathways leading to AMP production [194,195,196].

## 3. Conclusions

**Figure 11:**
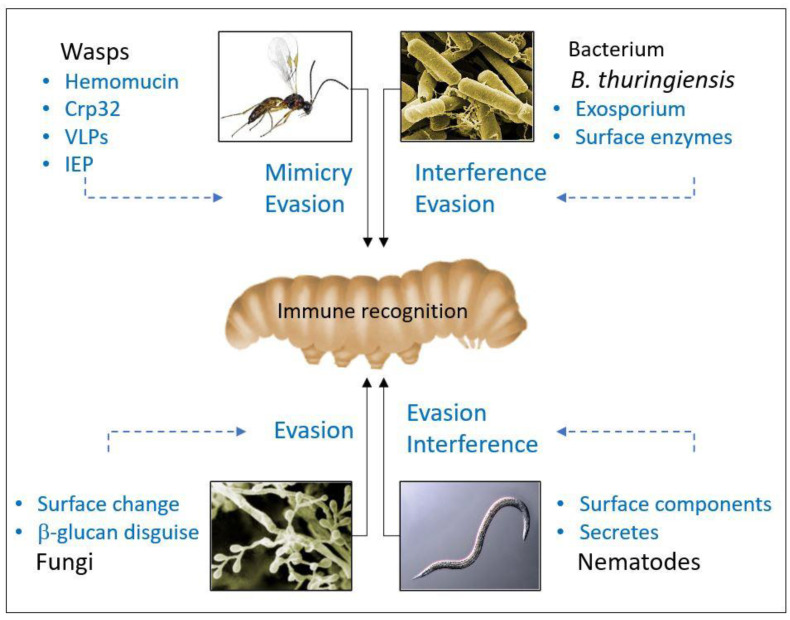
A schematic of the role of compounds at the surface of entomopathogens.

Beyond any consideration of the complex mechanisms established by entomopathogens, what clearly emerges from the specific literature is the central role that the body and cell surfaces play; certainly, escaping the control exerted by the discriminatory processes of the immune response on the presence of possible pathogens forms the basis of these organisms’ success. The fundamental processes of innate immunity are based on the possibility that pattern receptors recognize molecules exposed by the invader quickly and effectively, so it is essential that a potential pathogen can alter and/or mask surface compounds that can interact with the host PRRs, and thus its surface architecture and composition. As described in this review, all of the entomopathogens used as bioinsecticides have evolved sophisticated strategies aimed at drastic defusing of the immune response effector processes, but more conservatively, many of them have developed methods that allow them, in the earliest stages of the infection, to confuse the action of the recognition receptors, gaining essential time to prepare for a more drastic fight in the later stages (Figure 11).

Here, we have described the strategies of entomopathogens, such as molecular disguise, surface interaction with host molecules, removal by affinity of factors essential for the onset of defense processes, and the presence of faint envelopes releasable if recognized by the host cells; all these processes are aimed at allowing pathogens more time to prepare the next stages of the infection undisturbed, i.e., the final drastic actions that deeply alter the insect’s physiology and lead to its death. 

Besides, it is clear that an immunocompatible surface with antigens closely related to those of the host could be the best evasion strategy, i.e., the one with the highest compatibility with the host tissues and fluids, but this type of relationship certainly requires a long coevolution between the parasite and its specific host; this is the case of mutualistic relationships with a balance of advantages between the two parties, but it is clear that in this kind of relationship, the presence of a killer pathogen would certainly not be advisable.
